# Representation of a Monotone Curve by a Contour with Regular Change in Curvature

**DOI:** 10.3390/e23070923

**Published:** 2021-07-20

**Authors:** Yevhen Havrylenko, Yuliia Kholodniak, Serhii Halko, Oleksandr Vershkov, Oleksandr Miroshnyk, Olena Suprun, Olena Dereza, Taras Shchur, Mścisław Śrutek

**Affiliations:** 1 Department of Technical Mechanics and Computer Design Named after Prof. V.M. Naidysh, Dmytro Motornyi Tavria State Agrotechnological University, 18 B.Khmelnytsky Ave, 72312 Melitopol, Ukraine; yevhen.havrylenko@tsatu.edu.ua (Y.H.); yuliya.kholodnyak@tsatu.edu.ua (Y.K.); oleksandr.vershkov@tsatu.edu.ua (O.V.); olena.dereza@tsatu.edu.ua (O.D.); 2 Department of Electrical Engineering and Electromechanics Named after Prof. V.V. Ovharov, Dmytro Mo-tornyi Tavria State Agrotechnological University, 18 B.Khmelnytsky Ave, 72312 Melitopol, Ukraine; serhii.halko@tsatu.edu.ua; 3 Department of Electricity and Energy Management, Kharkiv Petro Vasylenko National Technical University of Agriculture, 19 Rizdviana Street, 61052 Kharkiv, Ukraine; 4 Department of Foreign Languages, Dmytro Motornyi Tavria State Agrotechnological University, 18 B.Khmelnytsky Ave, 72312 Melitopol, Ukraine; olena.suprun@tsatu.edu.ua; 5 Department of Cars and Tractors, Faculty of Mechanics and Energy, Lviv National Agrarian University, Volodymyr Great 1 Street, 80381 Dubliany, Ukraine; shchurtg@gmail.com; 6 Faculty of Telecommunications, Computer Science and Electrical Engineering, UTP University of Science and Technology in Bydgoszcz, 85-796 Bydgoszcz, Poland; mscislaw.srutek@utp.edu.pl

**Keywords:** interpolation, tangent line, curvature, area of location of the curve, contour, error, ellipse, B-spline

## Abstract

The problem of modelling a smooth contour with a regular change in curvature representing a monotone curve with specified accuracy is solved in this article. The contour was formed within the area of the possible location of a convex curve, which can interpolate a point series. The assumption that if a sequence of points can be interpolated by a monotone curve, then the reference curve on which these points have been assigned is monotone, provides the opportunity to implement the proposed approach to estimate the interpolation error of a point series of arbitrary configuration. The proposed methods for forming a convex regular contour by arcs of ellipses and B-spline ensure the interpolation of any point series in parts that can be interpolated by a monotone curve. At the same time, the deflection of the contour from the boundaries of the area of the possible location of the monotone curve can be controlled. The possibilities of the developed methods are tested while solving problems of the interpolation of a point series belonging to monotone curves. The problems are solved in the CAD system of SolidWorks with the use of software application created based on the methods developed in the research work.

## 1. Introduction

The technology of manufacturing products on numerical control machines (CNC) allows machining surfaces of arbitrary form. The program control for the CNC machine is created in automatic mode in the CAM system. The source data for the CAM system are represented by a three-dimensional computer model of the product formed in the CAD system (SolidWorks, AutoCAD, NXCAD etc.).

The accuracy of machining is determined by the accuracy of forming the three-dimensional model, the accuracy of determining the cutting tool trajectory by the CAM system and the accuracy of performing the programmed operations by the machine. The correct formation of the three-dimensional model is a necessary condition for manufacturing a high-quality product [1,2].

One of the most challenging problems in geometric modelling is creating models of surfaces bounding the items whose functional purpose is interaction with the environment (gas, liquid or loose materials). Examples of such products are automobile and aircraft hulls, working units of agricultural machinery and turbine blades. The main functional characteristic of the surfaces bounding such items is the laminar nature of their flow around by the environment [3,4,5].

The modelling technology in the CAD system is based on forming the surfaces on the basis of linear frameworks. Furthermore, the functional properties of the modelled surfaces were determined by the geometric characteristics of the curves comprising the framework. The laminar nature of the interaction of the surface with the environment was ensured by representing the elements of the framework by lines with regular changes in values of curvature and a minimum possible number of special points (according to the conditions of the problem). For a plane curve, these are the junction points of the convex and concave parts and the points with extreme curvature values [6].

In solving reverse engineering problems, when the given data for surface formation are represented as an array of points, the linear elements of the framework are formed by interpolating a point series. Thus, developing methods of interpolation for point series allowing the representation of the original curve with specified accuracy ensuring the given characteristics is a topical problem.

A smooth plane curve containing no special points will be called a curve with monotone curvature, or a monotone curve.

The problem of interpolating a point series specifying a monotone curve is solved by forming a contour—a curve consisting of sections of analytically determined curves joined at the reference points. At present, the most developed methods of forming contours are second-order curves, Bézier curves and B-splines [7,8,9,10,11].

Forming a smooth curve consisting of arcs of second-order curves [7] guarantees no uncontrolled emergence of inflection points along the contour; however, preventing the emergence of points with extreme values of curvature is impossible. Adjusting the shape of the contour is possible by changing the positions of the tangents to the contour at the reference points. With the positions of tangents fixed to the contour at the reference points, there is a possibility of locally adjusting the form of the curve while forming it by arcs of ellipses. The purpose of adjustment may be to equalize the radii of the curvature at junction points of the sections of the contour.

The use of Bézier curves [8,9] and B-splines [10,11] of third order and above when forming the contour provides greater control of its shape. B-spline is defined by the reference points, each of which has a corresponding conjugate function. It is represented by a composite curve, each segment of which determines a separate equation, the degree of which is equal to the degree of conjugate functions (k). Furthermore, at the junction points of the segments, the continuity of the derivative values to the k−1−th order inclusively is automatically ensured. The curve approximates the control polygon—a polygonal line connecting the reference points. The shape of the B-spline is adjusted by changing the position of the reference points. Control of curvature dynamics is carried out using the graph available in the CAD system. Increasing the quality of joining the sections of the contour requires increasing the degree of the spline equation resulting in an increase in the number of the reference points. As a result, local adjustments of the spline’s configuration and characteristics become more complex. It is difficult to achieve a monotone curvature along a B-spline interpolating a large number of reference points.

Modern CAD systems make it possible to form contours by a cubic B-spline, which automatically ensures the regularity of curvature values at the points of the contour. Control of the occurrence of special points within the sections of the contour can be manually performed by moving the phantoms of the control points specifying the spline with a cursor of a mouse. The need for manual adjustments depresses the possibility of using B-spline while forming contours with monotone change in curvature.

The work [12] proposes a method for forming a contour by sections representing a monotone curve with specified accuracy. The problem is solved in the following stages:

- Analysis of the reference point series is carried out, which results in identifying its sections that can be interpolated by a monotone curve;
- At the reference points, the positions of the tangent and the radii of the curvature are assigned, at which the problem of forming a monotone curve has a solution;
- The area of the possible location of the monotone curve interpolating sections of the point series is determined;
- Within the obtained area, a contour is formed, representing a monotone curve with specified accuracy.

The analysis of the reference point series is based on defining the radii of the adjacent circles (AC), each of which passes through three consecutive point series. Part of the point series along which the radii of the AC increase or decrease may be interpolated by a monotone curve along which the radii of curvature increase or decrease, respectively.

**Figure 1:**
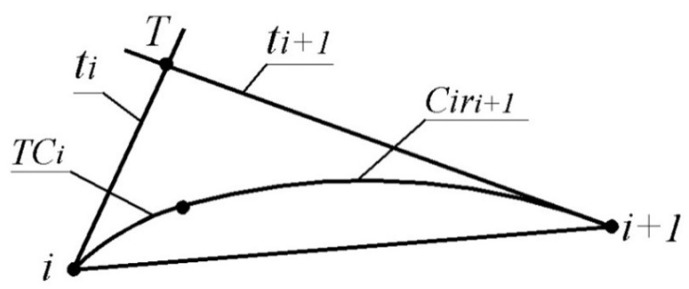
The lower boundary of the area of possible location of the monotone curve.

A criterion for the correct assignment of tangent lines of the monotone curve interpolating the point series along which the radii of curvature increase is the fulfilment of the ratio |i,T|<|T,i+1| (Figure 1), where T is the point of intersection of the tangents to the curve at nearly reference points ($t_{i}$ and $t_{i+1}$).

Based on this criterion, ranges of the positions of tangents are determined, at which a curve with monotone curvature can be formed. The final positions of tangents at the reference points are assigned to the centre of the obtained ranges.

Subsequently, the radii of curvature for which the problem of forming a monotone curve has a solution are assigned. A method for defining the ranges of possible values of curvature radii of a monotone curve interpolating a point series at fixed positions of tangents at the reference points is proposed in work [13]. For a curve along which the radii of the curvature increase, the minimum possible values of the radii of curvature at each point are defined proceeding from the condition that the radius of curvature equal to zero is assigned at the first point. The maximum radii of curvature are similarly defined by assigning the radius of curvature equal to infinity at the first point. The final values of the radii of the curvature at the reference points are assigned to the centre of the obtained ranges.

The positions of tangent lines and tangent circles assigned to the reference points define the area of the possible location of the monotone curve, interpolating a point series and possessing the specified characteristics at its points. The lower boundary of the area consists of the arc of the tangent circle at point *i*$\left(TC_{i}\right)$ and the arc of the circle which is tangent to $\left(TC_{i}\right)$ and line $t_{i+1}$ at point i+1$\left(Cir_{i+1}\right)$ (Figure 1). The upper boundary of the area is similarly defined based on the radius of curvature assigned at point i+1 and the position of line $t_{i}$.

Similar areas defined along the whole point series determine the possible location of the monotone curve interpolating the reference point series.

Work [12] proposes a method for forming arcs of circles whose radii increase in the same direction as the radii of the curvature of the monotone curve interpolating a point series. The disadvantage of this method is that the regularity of curvature values at the points at which the arcs of circles are conjoined is disturbed.

The aim of the present work was to develop a method for forming a contour with regular curvature change, representing a monotone curve with specified accuracy.

In order to achieve the aim of the research, the following objectives should be pursued:

- To develop a method for evaluating the accuracy with which the convex contour represents a curve with monotone curvature that can be used to interpolate the reference point series;
- To develop methods for forming a smooth convex curve with regular curvature interpolating with specified accuracy a point series by arcs of ellipses and B-spline;
- To compare the potential of the proposed methods in solving the problem of interpolating a point series.

## 2. Materials and Methods

To ensure the regularity of change in the curvature values along the contour at the joining points of its sections, it is necessary to provide common tangents and curvature values. Such a problem can be solved by using the sections of curves whose parametric number (the number of fixed parameters that unambiguously determine the geometric image) is not less than four. The problem of forming a regular contour representing a monotone curve with specified accuracy is investigated using curves with a corresponding parametric number, which is used in SolidWorks. These are parabola, ellipse and B-spline.

The accuracy with which the contour represents a monotone curve will be evaluated by the maximum absolute interpolation error, a value which cannot exceed the distance between the contour and the monotone curve.

The use of the curves listed above does not ensure a monotone change in curvature values along the contour, but makes it possible to control its convexity. Since the monotone curve is a convex curve, we will use the larger of the distances between the opposite boundaries (upper and lower) as the absolute interpolation error at each of the sections of the areas of the possible location of the monotone curve and convex curves, respectively.

**Figure 2:**
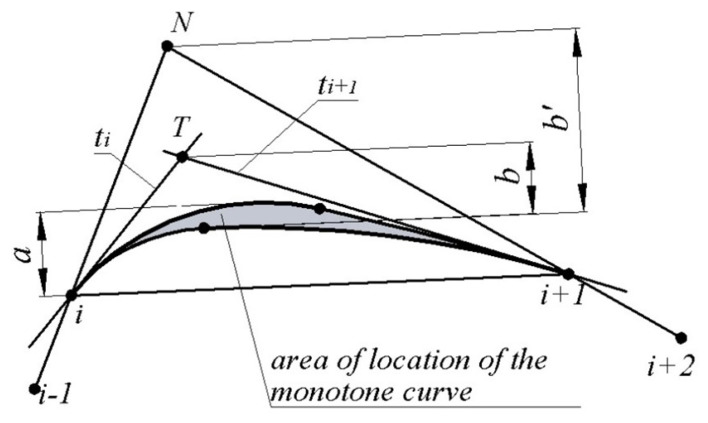
Area of the possible location of the section of the contour.

If the positions of the tangents to the contour have been assigned at the reference points, the area of possible location of its section is triangle i,i+1,*T* bounded by segment [i,i+1] connecting the successive reference points to tangents $t_{i}$ and $t_{i+1}$ (Figure 2).

In this case, the maximum interpolation error of section i…i+1 is estimated to be greater than *a* or *b*, where:

*a*—distance from the side of triangle [i,i+1] to the tangent to the upper boundary of the area of location of the section of the monotone curve which is parallel to segment [i,i+1];

*b*—distance from the apex of triangle *T* to the lower boundary of the area of location of the monotone curve.

If the positions of the tangents to the convex contour are unknown, the area of the possible location of the section of the contour i…i+1 can be defined as the triangle i,i+1,N, bounded by lines [i−1,i],[i,i+1],[i+1,i+2] (Figure 2). In this case, the error of interpolation of the sections is estimated to be greater than a or $b^{\prime }$, where $b^{\prime }$ is the distance from the lower boundary of the area of location of the monotone curve to the intersection point of lines [i−1,i],[i+1,i+2] (point N). The value of $b^{\prime }$ exceeds the value of *b*, and it will usually determine the error of interpolation by the contour at the reference points of which the positions of the tangents are not defined.

When forming a contour representing a monotone curve, it is appropriate to assign positions of the tangents at the reference points where a point series can be interpolated by a monotone curve [12].

Smooth joining of the sections can be achieved by forming them with a curved line, whose parametric number is not less than four. For section i…i+1, the two conditions applied to the curve are a passage through the fixed reference points *i* and i+1, and contingency with lines $t_{i}$ and $t_{i+1}$ produces the third and fourth conditions, respectively. In order to ensure the regularity of change in curvature along the contour, the parametric number of curves forming the contour must be larger than four.

The parametric number of curves specified by an algebraic equation can be defined by the number of coefficients of its equation. For a parabola whose general equation is $Ay^{2}+Bxy+Cx+Dy+1=0$ [14], the parametric number is equal to four. Therefore, the conditions of passage through two fixed points and contingency with two fixed lines completely specify the curve. Imposing additional conditions on the section of the parabola ensuring equal curvature values at the junction points of the sections of the contour is not possible.

The general equation of an ellipse has the following form:

(1) $$Ax^{2}+Bxy+Cy^{2}+Dx+Ey+1=0$$

The number of coefficients of the equation determines the parametric number of the curve equal to five. Let us demonstrate how the possibility of imposing five conditions on the section of the curve ensures its regularity.

Once the tangents to the monotone curve are assigned at the reference points, four of the five conditions specifying each of the sections of the contour are defined.

There is a set of ellipses meeting the four indicated conditions. To extract an unambiguous solution from the set, it is necessary to impose a fifth condition on the arc of the ellipse. As a fifth condition, let us define that the ellipse passes through another fixed point *M*. In order to reduce the number of calculations required, let us assign point *M* on the median of triangle i,i+1,T. At the same time, to ensure the minimum error with which the arc of the ellipse replaces the section of the monotone curve, point *M* is assigned within the area of the possible location of the monotone curve.

The conditions of the passage of the arc of the ellipse through points $1(x_{1}$,$y_{1})$ and $2(x_{2}$,$y_{2})$ bounding the area and point $M(x_{M}$, $y_{M})$ define the following correlations:

(2) $$Ax_{1}^{2}+Bx_{1}y_{1}+Cy_{1}^{2}+Dx_{1}+Ey_{1}+1=0$$

(3) $$Ax_{2}^{2}+Bx_{2}y_{2}+Cy_{2}^{2}+Dx_{2}+Ey_{2}+1=0$$

(4) $$Ax_{M}^{2}+Bx_{M}y_{M}+Cy_{M}^{2}+Dx_{M}+Ey_{M}+1=0$$

By differentiating Equations (2) and (3), we obtain:

(5) $$2Ax_{1}+B\left(y_{1}+x_{1}y_{1}{}^{\prime }\right)+2Cy_{1}y_{1}{}^{\prime }+D+Ey_{1}{}^{\prime }=0$$

(6) $$2Ax_{2}+B\left(y_{2}+x_{2}y_{2}{}^{\prime }\right)+2Cy_{2}y_{2}{}^{\prime }+D+Ey_{2}{}^{\prime }=0$$

If the tangent lines at points 1 and 2 are determined by the equations $y=xa_{1}+b_{1}$ and $y=xa_{2}+b_{2}$, respectively, the values of the derivatives of the function in Equations (5) and (6) are defined as $y_{1}{}^{\prime }=a_{1}$, $y_{2}{}^{\prime }=a_{2}$.

By solving the system of Equations (2)–(6) we define the values of coefficients A,B,C,D,E and obtain an equation that defines the first section of the curve of form (1).

In order to determine the value of the second derivative of the function defining the ellipse at point 2, it is necessary to differentiate Equation (1) twice and substitute the coordinates of point $2{(}_{x2}$,${}_{y2})$. By extracting the second derivative of the function from the equation, we obtain:

(7) $$y^{\prime \prime }=-\frac{2Bx_{2}+2Cy_{2}+E}{2A+4B_{y}{}^{\prime }+2C{\left(y{}^{\prime }\right)}^{2}{}^{\prime }}\;$$

where:

$$y^{\prime }=-\frac{2Bx_{2}+2Cy_{2}+E}{2Ax_{2}+2By_{2}+D}\;$$

For the regular joining of the first and second sections of the curve, in addition to the common tangent at point 2, it is sufficient to ensure the equality of the second derivatives of the functions specifying the arcs of ellipses at that point.

To determine the coefficients of the equation specifying the ellipse that forms the second section of the curve, it is necessary to solve a system of five equations:

- Equations (3) and (6);
- Equation (7) with substituted values of $y^{\prime \prime }$ defined by coefficients of the equation specifying the arc of the ellipse for the first section of the contour;
- Equations defining characteristics of the contour at point 3:

$$Ax_{3}^{2}+Bx_{3}y_{3}+Cy_{3}^{2}+Dx_{3}+Ey_{3}+1=0$$

$$2Ax_{3}+B\left(y_{3}+x_{3}y_{3}{}^{\prime }\right)+2Cy_{3}y_{3}{}^{\prime }+D+Ey_{3}{}^{\prime }=0$$

**Figure 3:**
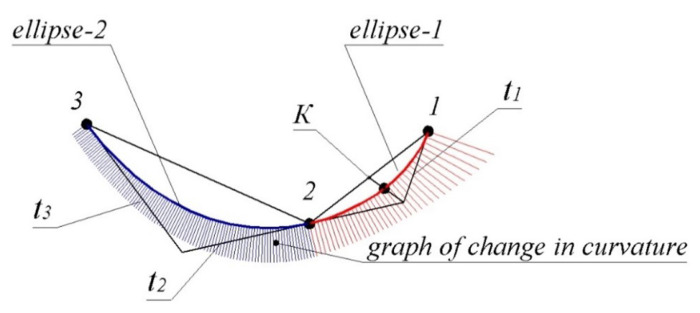
Forming a contour by arcs of ellipses.

The first and second sections of the contour, the characteristics of which are calculated according to the method described above, are shown in Figure 3.

The contour was formed in the CAD system SolidWorks with the use of a specially developed software module. The given data are represented by a point series belonging to a monotone curve interpolation which was studied in the work [12]. The graph of curvature values along the contour formed with the CAD system demonstrates the regularity of joining of the sections. The maximum absolute interpolation error was 0.8735 mm in the first section and 0.6328 mm in the second section.

The maximum deflection of the contour from the boundaries of the areas of the possible location of the monotone curve in each of the sections was 0.0121 and 0.0053 mm, respectively. The monotone change in curvature values along the arcs of ellipses forming the sections of the curve determined their location within the area of the location of the monotone curve. However, the ways to predict the absence of points of extreme curvature within the arc of the ellipse specified by the characteristics at boundary points are unknown.

After determining the coefficients defining the equations of the ellipse, which represent the second section of the contour, the equations of the third and subsequent sections were similarly determined. Furthermore, the fifth condition defining the configuration of each of the arcs of ellipses is the equality of second derivatives with the previous section of the contour at the point of junction.

The disadvantage of the proposed method for forming the contour is the lack of possibility to locally adjust its form. The reconfiguration of the first section will change the configuration of all other sections. The reason for this deficiency is the imposition of as many conditions as possible on each of the sections of the contour.

The possibility to locally adjust the form of the sections is a major advantage of forming the contours by B-spline. The degree of the equation specifying the B-spline determines the order of the derivative functions whose values coincide at the junction points of the sections of the spline [15]. The third-degree equation specifying the sections of B-spline that is used in the CAD system SolidWorks automatically ensures the regularity of change in curvature values along the contour of arbitrary configuration.

The interpolation of a point series in the CAD system is performed interactively—the reference points are sequentially indicated with the cursor. If the reference point series can be interpolated by a convex curve, then the B-spline has the form of a convex contour, the number of sections of which is minimal—as each section of the contour is formed by one section of the B-spline.

The specified sequence of commands is easily implemented by the software application, providing the automatic formation of a convex contour in the CAD system SolidWorks.

The imposition of the tangency condition with fixed straight lines on the B-spline requires an increase in the parametric number of curves by increasing the number of spline sections. This could lead to a disturbance of the convexity of the contour. In CAD systems, the convexity of B-spline can be achieved interactively by adjusting the position of the vertices of the specifying polygon. With a large number of reference points and tangents assigned to them, the task becomes excessively laborious.

Creating an application that automatically generates a convex regular B-spline specified by a sequence of reference points and positions of tangents at those points is a complex task. Examples of solving this problem are unknown. When forming a B-spline representing a monotone line with specified accuracy, the simplest and most effective solution is to increase the number of reference points that can be interpolated by a monotone curve.

## 3. Results and Discussion

**Figure 4:**
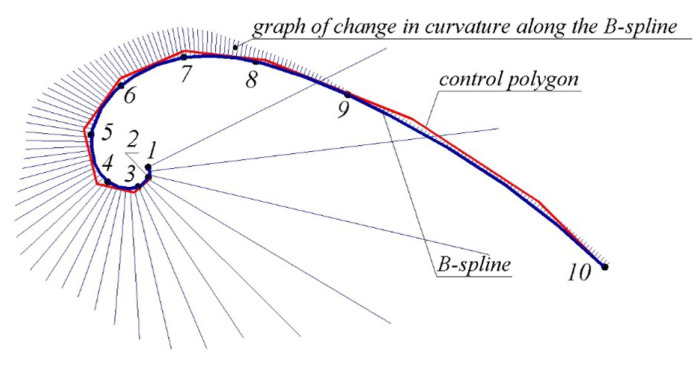
B-spline specified by the coordinates of the reference points.

Figure 4 shows a cubic non-periodic B-spline interpolating a point series consisting of 10 points created in the CAD system SolidWorks. The sequence of points that was used in [12] was taken as the reference point series to determine the area of location of the monotone curve.

The spline consists of nine segments whose configuration is determined by the 12 specifying points. The graph of change in the curvature values along the B-spline formed in the CAD system allows determining whether it has special points. The sections of the spline contain seven points with extreme values of curvature in total.

Table 1 presents the characteristics of the reference point series and error values of its interpolation by B-spline, where:

1. $x_{i}$, $y_{i}$—coordinates of the reference points;
2. $h_{i}=\left|i,i+1\right|$—lengths of segments connecting the reference points;
3. $\delta_{i}$—maximum absolute error of interpolation of a point series by a monotone curve;
4. The width of the area of the possible location of the segments of the convex contour, which is equal to:
  - Distance $H_{i}$ from section [i,i+1] to the intersection point of lines (i−1,i) and (i+1,i+2);
  - Distance $H_{i}^{t}$ from section [i,i+1] to the intersection point of tangents $t_{i}$ and $t_{i+1}$;
5. The absolute error with which a monotone curve is represented by sections of the contour is:
  - $\Delta_{i}$, when the curve is specified by a point series;
  - $\Delta_{i}^{t}$ when the curve is specified by a point series and by the location of the tangents at the reference points;
6. $f_{i}$—the maximum distance from the formed B-spline to the furthest boundary of the area of possible location of the monotone curve.

Meeting the condition of tangency with ten fixed tangents to the monotone curve assigned at the reference points required increasing the parametric number of the spline by increasing the number of its segments. The resulting B-spline consists of 14 segments whose configuration is determined by 16 specifying points. By adjusting the configuration of the B-spline using the tools of the CAD system SolidWorks “Add Tangency Control”, “Add Curvature Control” and “Spline Handles”, it was possible to achieve the convexity of the entire contour with no more than one special point on sections 6…7, 7…8, 8…9 and 9…10 of the curve.

The resulting B-spline was a convex curve that contains thirteen points with extreme values of curvature. On sections containing special points, the spline was located outside the area of a possible location of the monotone curve with a maximum deflection of 0.1182 mm on sections 3…4. On sections 6…7, 7…8, 8…9, 9…10, where the curvature values change monotonously, the spline is located within the area of the location of the corresponding sections of the monotone curve.

In Table 2, the error values of the interpolation of a point series by the arcs of ellipses are presented. For the correctness of the comparison of the characteristics of the contours formed by arcs of various curves, the same point series that was used in interpolation by B-spline (Table 1) was taken as source data.

The error of interpolation by a contour consisting of arcs of ellipses was analogous to the interpolation error of the B-spline which has common tangents with the monotone curve at the reference points. The maximum interpolation error of the entire point series was determined by the section of the contour where the values of $\Delta_{i}$ or $\Delta_{i}^{t}$ are the greatest. These will be the longest sections or the ones where the curvature of the contour is extreme. For the contours, represented in Table 1 and Table 2, it is section 9 for which $\Delta_{i}=14.8909$, $\Delta_{i}^{t}=5.8393$. The values of $\Delta_{i}$ and $\Delta_{i}^{t}$ are determined by the configuration of the reference point series and do not depend on the curve sections of which the contour is formed.

The actual deflection of the convex curve from the boundaries of the area of the possible location of the monotone curve ($f_{i}$) depends on the characteristics of the contour on each section. In the case of the monotonous change in curvature along the contour and common tangents with the monotone curve on its boundaries, the deflection of the contour from the boundaries of the area of location of the monotone curve does not exceed the width of that region. If the section of the contour contains points with extreme values of curvature, ways to determine in advance the position of the contour within the area of the convex curve are unknown. The value of $f_{i}$ is determined by the characteristics of the curve forming the sections of the contour and by the location of tangents to the contour at the reference points.

It can be guaranteed that the error in the representation of a monotone curve by a convex curve does not exceed the maximum distance between the boundaries of their possible location. This error can be arbitrarily reduced by increasing the number of reference points.

Table 3 presents the characteristics of a point series consisting of 19 points and error values of its interpolation by a contour formed by the arcs of ellipses. The point series consists of 10 reference points whose coordinates are presented in Table 1 and Table 2, and nine intermediate points. The indicated 19 points can be interpolated by a monotone curve. The position of intermediate points and the position of lines tangent to the monotone curve at 19 points were determined by a method proposed in [12].

By reducing the distances between the reference points by approximately two-fold, the guaranteed interpolation error $\Delta_{i}^{t}$ was reduced by more than four times.

Reducing the interpolation error $\Delta_{i}$ by increasing the number of reference points can be demonstrated by the example of replacing the involute of the circle with a convex B-spline.

The involute of the circle is a monotone curve along which the curvature values are reduced. Its parametric equation has the following form [16]:

x=rcosφ+rφsinφ;

y=rcosφ−rφsinφ,

where, *r*—radius of the circle (evolute) defining the involute; φ—the angle corresponding to the arc of the involute.

The coordinates of the points belonging to the involute of the circle and making up the original point series for the interpolation by B-spline were obtained for the step of changing the values of the parameter $\Delta \varphi =\varphi_{i+1}-\varphi_{i}=1^{\circ }$ starting from the point determined by the value of the parameter $\varphi_{i}=0$.

**Figure 5:**
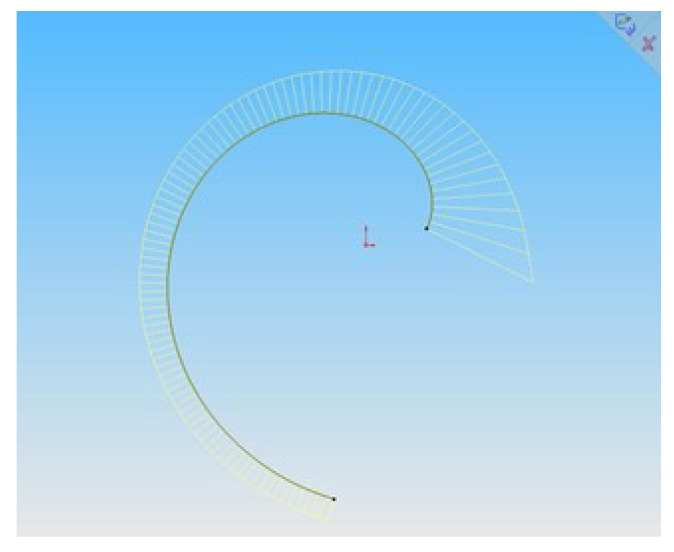
Representation of the involute of the circle by B-spline.

We assigned 270 points on the involute of the circle, which were interpolated by B-spline. The characteristics of the point series are given in Table 4, and the B-spline interpolating the point series is shown in Figure 5.

The values of the interpolation of the first 10 points and the last of the point series by a convex contour at points of which the position of the tangents is not defined $\left(\Delta_{i}\right)$ are presented in Table 4.

With the use of a software developed by us, the obtained point series consisting of 270 points is interpolated by a cubic B-spline which is imported into the CAD system SolidWorks. Figure 5 shows a section of the involute and a graph of change in the curvature along it.

The accuracy with which the B-spline represents the evolution of of the circle is provided by a graph of change in curvature values along the contour, which are defined by the CAD system tools as monotone.

## 4. Conclusions

This work investigated the possibility of forming a smooth regular contour, which with specified accuracy, represents a monotone curve. The curves whose sections can form a regular contour do not provide a monotonous change in curvature values along the contour, but ensure the control of its convexity. Therefore, it is proposed that the largest distance between the areas of the possible location of the monotone and the convex curves is used, specified by the same point series as an absolute error on each of the sections.

The main criteria for evaluating the computational efficiency of interpolation methods are error, stability and convergence. The interpolation method is stable if negligible errors of the source data lead to minor changes in the result. The use of stable interpolation methods reduces the accuracy requirements of the source data and make it possible to adjust them. The interpolation convergence consists in reducing the error by increasing the number of reference points. When interpolating a point series of arbitrary configuration consisting of a large number of points, most of the known methods do not ensure the convergence and stability of the solution. The reason for this deficiency of interpolation methods is the uncontrolled emergence of inflection points of convexity/concavity of the interpolating curve.

The maximum absolute interpolation error is estimated by the value that cannot be exceeded by the distance between the contour and any monotone curve interpolating the reference point series. Such an error is determined by the width of the area bounded by a sequence of triangles. The sides of the triangles belong to lines connecting the reference points or lines tangent to the monotone curve. By assigning intermediate points, the width of the area of location of the curve is reduced.

The main advantage of the proposed method over known interpolation methods is the possibility to form the contour within a bounded spatial domain. The domain was defined by the expected characteristics of the reference curve. The most important of these characteristics is the minimum (based on the configuration of the reference point series) number of special points of the curve.

The methodology for estimating the error of existing interpolation methods is as follows. A point series is assigned on the reference curve. After interpolating the point series, the deflection of the interpolating curve from the reference curve is determined. The error with which the interpolating curve represents the reference curve is estimated as the maximum deflection between the specified curves. When solving other tasks, the engineer operates on the premise that the order of error of interpolation will be identical with the test examples. Generally, when the reference curve is not known, such an assumption might be wrong.

Defining the area of the possible location of the reference curve not only allowed to determine the absolute interpolation error, but also to arbitrarily decrease this error by increasing the number of reference points or assigning intermediate points by the method proposed in [12].

The convergence and stability of the proposed method are ensured by the preliminary analysis of the configuration of the reference point series and formation of the contour based on sections of the point series which can be interpolated by a monotone curve.

The stability of the developed method to changes of reference conditions is ensured by forming a contour within the area of the possible location of the convex parts of the reference curve. With any adjustment of the positions of the reference points, the distance from the segments connecting the reference points to the boundaries of the possible location of the corresponding sections of the reference curve remains limited.

The consistent localization of the area of the location of the curve within its original boundaries guarantees the convergence of the interpolation process. Adding each additional point brings the boundary of the area closer to the accurate solution, namely to a monotone curve with a given set of characteristics.

The number of sections of point series that can be interpolated by a monotone curve, as well as the number of reference points that make up each of these sections, does not affect the convergence or stability of interpolation.

Any curve can be considered to consist of monotone parts. Thus, the assumption that if a sequence of points can be interpolated by a monotone curve, then the reference curve on which these points were assigned is monotone, allows implementing the proposed approach for estimating the error of interpolation of a point series of arbitrary configuration.

Defining the area of the possible location of the reference curve enables developing methods for forming contours by arcs of various curves, the convexity of which can be controlled. Based on the proposed criteria for estimating the interpolation error, methods for forming convex regular contours by arcs of the ellipses and B-spline were developed. The methods were implemented in a software application that automatically creates contours in the CAD system SolidWorks. The method for forming a contour by arcs of an ellipse is based on the derivation of equations specifying ellipses and the determination of parameters by these equations, which make it possible to form sections of the curve in the SolidWorks system. Such parameters are the coordinates of the points bounding the contour, the positions of the tangent lines to the monotone curve at these points and the coordinates of another point belonging to the ellipse.

The application of forming contours by B-spline ensures its passage through a sequence of points specified by their coordinates.

The method for forming contours by arcs of ellipses takes into account the positions of tangents to the contour and provides for greater interpolation accuracy. The method for forming contours by B-spline requires fewer calculations and is more easily executed in the form of software.

The possibilities of the method for forming curves by B-spline were investigated while solving the following problems:

- Interpolation of a point series consisting of ten points specifying a monotone curve;
- Interpolation of a point series belonging to the involute of the circle.

This study proved that an arbitrarily small interpolation error can be achieved by reducing the distances between the reference points belonging to the monotone curve by increasing their number. The sequence of interpolated points can be increased either by refining the given data or by assigning intermediate points according to the methodology proposed in [12].

The developed methods are appropriate for modelling linear elements of the frame of functional surfaces with improved aerodynamic and hydrodynamic properties, for which the minimum number of special points is the main criterion of the quality of the obtained solution.

The advantages of the methods proposed in this work over the method developed in [12] for presenting a monotone curve by a contour formed by arcs of circles is the regular change in curvature values. The advantage of the method proposed in [12] is the monotonous change in curvature values along the contour. Both of these characteristics are important for modelling functional surfaces. While solving particular problems, it does not seem possible to prioritise one of the characteristics in advance as it will depend on the conditions of the problem. Such conditions are the speed of the flow of the environment along the surface and the physical characteristics of the environment (density, viscosity etc.).

The influence degree of the quality of joining the sections of the curve and the conformity of the change in curvature values along the curve can be determined by experiment.

Increasing the number of interpolated points is a universal method of increasing the accuracy with which a regular contour represents a monotone curve and reducing the difference in curvature values at points where arcs of circles are joined.

## Figures and Tables

**Table 1:** Characteristics of the reference point series and error values of its interpolation by B-spline.

*i*	$x_{i}$	$y_{i}$	$h_{i}$	$\delta_{i}$	$H_{i}$	$H_{i}^{t}$	$\Delta_{i}$	$\Delta_{i}^{t}$	$f_{i}$
1	0	0	5.28	0.0140	3.1408	1.6068	2.4075	0.8735	0.1500
2	0.21	−5.28	7.20	0.0078	4.4045	1.2234	3.8139	0.6328	0.0824
3	−5.11	−10.13	15.57	0.0218	11.7259	4.2358	9.8524	2.3623	0.1440
4	−20.48	−7.65	25.24	0.0595	18.3244	8.1017	14.3855	4.1628	0.6637
5	−28.68	16.23	28.82	0.0629	12.6391	4.7848	10.4325	2.5782	0.4586
6	−13.87	40.95	34.90	0.0922	10.4670	5.2981	7.8280	2.6591	0.3054
7	17.95	55.28	36.29	0.2184	6.8370	3.2593	5.2685	1.6908	0.1534
8	54.24	53.04	49.65	0.2257	6.7010	2.6676	5.4764	1.4320	0.1712
9	100.94	36.19	155.96	1.1281	19.7329	10.6813	14.8909	5.8393	1.5414
10	230.38	−50.82	-	-	-	-	-	-	-

**Table 2:** Characteristics of the reference point series and error values of its interpolation by a contour formed by arcs of ellipses.

*i*	$x_{i}$	$y_{i}$	$h_{i}$	$\delta_{i}$	$H_{i}^{t}$	$\Delta_{i}^{t}$	$f_{i}$
1	0	0	5.28	0.0140	1.6068	0.8735	0.0121
2	0.21	−5.28	7.20	0.0078	1.2234	0.6328	0.0053
3	−5.11	−10.13	15.57	0.0218	4.2358	2.3623	0.0714
4	−20.48	−7.65	25.24	0.0595	8.1017	4.1628	0.1642
5	−28.68	16.23	28.82	0.0629	4.7848	2.5782	0.0415
6	−13.87	40.95	34.90	0.0922	5.2981	2.6591	0.0661
7	17.95	55.28	36.29	0.2184	3.2593	1.6908	0.2948
8	54.24	53.04	49.65	0.2257	2.6676	1.4320	0.3041
9	100.94	36.19	155.96	1.1281	10.6813	5.8393	1.3420
10	230.38	−50.82	-	-	-	-	-

**Table 3:** Characteristics of the point series and error values of its interpolation by a contour formed by arcs of ellipses.

*i*	$x_{i}$	$y_{i}$	$h_{i}$	$\delta_{i}$	$H_{i}^{t}$	$\Delta_{i}^{t}$	$f_{i}$
1	0	0	2.74	0.0021	0.3770	0.1955	0.0091
2	0.85	−2.61	2.74	0.0014	0.3909	0.1972	0.0085
3	0.21	−5.28	3.65	0.0018	0.2660	0.1373	0.0031
4	−2.05	−8.14	3.65	0.0011	0.3289	0.1695	0.0039
5	−5.11	−10.13	8.01	0.0026	0.9969	0.5291	0.0523
6	−13.09	-10.74	8.01	0.0028	0.9623	0.4704	0.0861
7	−20.48	−7.65	13.23	0.0071	2.2027	1.0666	0.1481
8	−28.32	3.00	13.23	0.0075	1.7039	0.8308	0.0721
9	−28.68	16.23	14.58	0.0077	1.1985	0.5771	0.0014
10	−23.19	29.74	14.58	0.0084	1.1187	0.5399	0.0011
11	−13.87	40.95	17.65	0.0115	1.2179	0.5808	0.0102
12	0.94	50.54	17.65	0.0121	1.3186	0.6311	0.0092
13	17.95	55.28	18.26	0.0268	0.8529	0.4051	0.2185
14	36.20	55.82	18.26	0.0271	0.7170	0.3416	0.2651
15	54.24	53.04	24.86	0.0281	0.8677	0.4574	0.2861
16	78.05	45.89	24.86	0.0296	0.4971	0.2600	0.7652
17	100.94	36.19	78.17	0.1516	2.9496	1.4995	1.2075
18	168.69	−2.81	78.17	0.1682	2.3530	1.1534	1.4265
19	230.38	−50.82	-	-	-	-	-

**Table 4:** Values of the interpolation of a point series by a convex contour.

*i*	$x_{i}$	$y_{i}$	$h_{i}$	$\Delta_{i}$
1	35	0	0.5$\;\times \;10^{-4}$	-
2	35	0.6$\;\times \;10^{-4}$	1.60 $\;\times \;10^{-2}$	1.30$\;\times \;10^{-4}$
3	35.02	0.5$\;\times \;10^{-3}$	2.67$\;\times \;10^{-2}$	2.289$\;\times \;10^{-4}$
4	35.05	1.7$\;\times \;10^{-3}$	3.73$\;\times \;10^{-2}$	3.233$\;\times \;10^{-4}$
5	35.09	3.97$\;\times \;10^{-3}$	4.80$\;\times \;10^{-2}$	4.169$\;\times \;10^{-4}$
6	35.13	7.74$\;\times \;10^{-3}$	5.86$\;\times \;10^{-2}$	5.103$\;\times \;10^{-4}$
7	35.19	1.34$\;\times \;10^{-2}$	6.93$\;\times \;10^{-2}$	6.036$\;\times \;10^{-4}$
8	35.26	2.12$\;\times \;10^{-2}$	7.97$\;\times \;10^{-2}$	6.968$\;\times \;10^{-4}$
9	35.34	3.16$\;\times \;10^{-2}$	9.06$\;\times \;10^{-2}$	7.90$\;\times \;10^{-4}$
10	35.43	4.51$\;\times \;10^{-2}$	10.13$\;\times \;10^{-2}$	8.83$\;\times \;10^{-4}$
…	…	…	…	…
261	−162.49	−6.89	2.777	2.423$\;\times \;10^{-2}$
262	−162.95	−9.63	2.787	2.433$\;\times \;10^{-2}$
263	−163.36	−12.39	2.798	2.442$\;\times \;10^{-2}$
264	−163.73	−15.16	2.809	2.452$\;\times \;10^{-2}$
265	−164.73	−17.95	2.819	2.461$\;\times \;10^{-2}$
266	−164.32	−20.76	2.830	2.470$\;\times \;10^{-2}$
267	−164.54	−23.58	2.841	2.479$\;\times \;10^{-2}$
268	−164.71	−26.42	2.851	2.489$\;\times \;10^{-2}$
269	−164.83	−29.27	2.862	2.498$\;\times \;10^{-2}$
270	−167.91	−32.13	2.873	-

## Data Availability

The data presented in this study are available on request from the corresponding author.
